# Novel Mechanisms for Post-Transplant Maintenance Therapy in Acute Myeloid Leukemia

**DOI:** 10.3389/fonc.2022.892289

**Published:** 2022-07-12

**Authors:** Steven A. Manobianco, Tara Rakiewicz, Lindsay Wilde, Neil D. Palmisiano

**Affiliations:** ^1^ Thomas Jefferson University Hospital, Jefferson University Hospitals, Philadelphia, PA, United States; ^2^ Department of Medical Oncology, Division of Hematologic Malignancy and Stem Cell Transplantation, Philadelphia, PA, United States

**Keywords:** AML – acute myeloid leukaemia, maintanance, novel treatment, stem cell transplant (SCT), post-transplant

## Abstract

Allogeneic stem cell transplantation has improved survival for patients with acute myeloid leukemia (AML), especially for patients with disease at high risk of relapse. However, relapse remains the most common cause of treatment failure and death in the post-transplant period. Maintenance therapy, an extended course of treatment after achieving remission to reduce the rate of relapse, is an important component of the treatment of various hematologic malignancies; however, its role in the treatment of AML is far less well-defined. Recently, there has been significant interest in the use of novel therapeutic agents as maintenance therapy after allogeneic stem cell transplant, utilizing new mechanisms of treatment and more favorable toxicity profiles. In this review, we will discuss the mechanistic and clinical data for post-transplant maintenance therapies in AML. Then, we will review several emergent and current clinical trials which aim to incorporate novel agents into maintenance therapy regimens.

## Introduction

Since its initial description in the 1950s, allogeneic stem cell transplantation has improved survival for patients with acute myeloid leukemia (AML), especially for patients with disease at high risk of relapse (1). Despite this life-saving advancement, relapse remains the most common cause of treatment failure and death in the post-transplant period, representing the primary cause of death for more than half of transplant recipients depending on the type of transplant received (2). Survival after relapse remains poor, with less than 25% of patients alive at 1 year post-relapse and less than 20% at 2 years (3, 4). These figures underscore the importance of identifying treatments to decrease rates of relapse and improve post-transplant survival.

Maintenance therapy, an extended course of treatment after achieving remission to reduce the rate of relapse, is an important component of the treatment of various hematologic malignancies including acute lymphoblastic leukemia; however, its role in the treatment of AML is far less well-defined (5). Post-transplant maintenance for AML dates back to the 1960s, when chemotherapeutic agents and/or early immunotherapies such as interferon were trialed (6, 7). The use of these agents was not broadly adopted due to both their high degree of toxicity and the unclear survival benefit (8). More recently, there has been a groundswell of interest in the use of novel therapeutic agents as maintenance therapy after allogeneic stem cell transplant, leveraging new understanding and identification of genetic mutations, epigenetic influences, and cell-signaling pathways which play critical roles in the behavior of leukemic cells combined with the more favorable toxicity profiles of these agents (7, 8).

In this review, we will discuss the mechanistic and clinical data for post-transplant maintenance therapies in AML. Then, we will review several emergent and current clinical trials which aim to incorporate novel agents into maintenance therapy regimens. Many of these key studies are listed in **Table 1**.

**Table 1:** Select Studies of Novel Therapeutics as Post-Transplant Maintanence Therapy in AML.

Drug Class	Trial (Year)	Reference	Phase	Drug/Dose Schedule	Patients (N)	Age Range of Patients	Primary Outcome
Tyrosine Kinase Inhibitors (TKIs)	Xuan (2020)	(9)	3	Sorafenib 400 mg twice daily from post-transplant days 30 to 180 vs control (no maintanence)	202	26-43	1-year relapse: 7.0% vs 25.0% (p=0.0010)
	Burchert (2020)	(10)	2	Sorafenib 400 mg daily titrated to 400mg twice daily up to 24 months vs placebo	83	18-75	2-year RFS: 85% vs 53% (p=0.002)
	Maziarz (2021)	(11)	2	Midostaurin 50 mg twice daily in 12 4-week cycles + standard of care (SOC) vs SOC	60	18-70	18-month RFS: 89% vs 76% (p = 0.27)
	NCT03690115	Not Applicable	2	Ponatinib 30 mg daily	Not Applicable	18-70	2-year relapse: pending
	NCT02997202	(12)	3	Gilteritinib daily (dose not specificed) vs placebo	Not Applicable	>18	7-year RFS: pending
	NCT02400255	Not Applicable	2	Crenolanib 100 mg three times daily for up to 728 days	Not Applicable	>18	2-year PFS: pending
	Sandmaier (2018)	(13)	1	Quizartinib 40 mg daily or 60mg daily for up to 24 28-day cycles	13	23-61	Tolerance: 5 patients completed 24 cycles
Histone Deacetylase Inhibitors (HDACi)	Bug (2017)	(14)	1/2	Panobinostat 20 mg three times weekly or 30mg three times weekly every second week	42	21-71	Tolerance: 22 (52%) received 12 months of treatment as planned
	NCT04326764	Not Applicable	3	Panobinostat 20 mg three times weekly every second week	Not Applicable	18-70	5-year OS: pending
IDH-1 and IDH-2 Inhibitors	Fathi (2020)	(15)	1	Enasidenib 50 mg daily or 100 mg daily in 28-day cycles	16	31-76	Tolerance: 3 (18%) patients discontinued study treatment
	NCT03728335	Not Applicable	1	Enasidenib (dose not specified) daily in 28-day cycles for up to 24 cycles	Not Applicable	>18	Incidence of AEs: pending
	NCT04522895	Not Applicable	2	Enasidenib 100 mg daily in 28-day cycles for up to 12 cycles	Not Applicable	>18	Incidence of AEs: pending
	NCT03564821	Not Applicable	1	Ivosidenib 500 mg daily in 28-day cycles with dose escalation or de-escalation after cycle 1	Not Applicable	>18	MTD: pending
Azacitidine (AZA)	de Lima (2010)	(16)	1	AZA 16 to 40 mg/m2 for 5 days in 28- to 30-day cycles	45	24-73	Optimal AZA dose: 32mg/m2 given for 4 cycles
	Guillaume (2019)	(17)	2	AZA 32 mg/m2 subcutaneously daily for 5 days for up to 12 28-day cycles, with esclated doses of donor lymphocyte infusions	30	18-70	Median time to relapse: 7 months (2.5–58); 2-year relapse: 27.6% (CI 95% = 12.8–44.6)
	Craddock (2016)	(18)	1/2	AZA 36 mg/m2 subcutaneously daily for 5 days for up to 12 28-day cycles	37	40-71	Tolerance: 31 patients completed 3 or more cycles of AZA
	Oran (2020)	(19)	3	AZA 32 mg/m2 subcutaneously daily for 5 days for up to 12 28-day cycles	93	19-75	RFS: 2.07 years (AZA) vs 1.28 years (control) (P = .43)
Oral AZA (CC-486)	de Lima (2018)	(20)	1/2	CC-486 200 mg or 300 mg once daily for 7 days per cycle or CC-486 150mg or 200mg daily for 14 days per cycle	30	28-80	1-year RFS: 72% with 14-day dosing vs 54% with 7-day dosing
AZA + Venetoclax	NCT04161885	Not Applicable	3	AZA (dose not specified) daily for 5 days with venetoclax (dose not specified) daily for 28 days for up to 6 28-day cycles	424	>18	MTD and RFS: pending
	NCT04128501	Not Applicable	2	AZA and venetoclax combination therapy (dosing and intervals not specified)	125	18-75	RFS: pending
Decitabine (DAC)	Pusic (2015)	(21)	1	DAC 5, 7.5, 10, and 15 mg/m2/day for 5 days for up to 8 6-week cycles	24	21-68	MTD: was not reached
DAC + Venetoclax	Wei (2020)	(22)	1	DAC 15 mg/m2/day for 3 days with venetoclax 200 mg daily for 21 days	6	>18	2-year OS and 2-year EFS: 83%
	Wei (2021)	(23)	2	DAC 15 mg/m2/day for 3 days with venetoclax 200 mg daily for 21 days for up to 10 2-month cycles	20	21-74	2-year OS: 85.2%; EFS: 84.7%
Immune Checkpoint inhibitors	Reville (2017)	(24)	2	Nivolumab 3 mg/kg intravenously every 2 weeks	15	31-71	Recurrence free survival: 8.48 months (95% CI: 2.14–NE)
	NCT02846376	Not Applicable	1	Nivolumab or ipililumab or combination (dosing and intervals not specified)	8	18-70	Tolerance and toxicity: pending

SOC, Standard of care; RFS, relapse free survival; PFS, progression free survival; OS, overall survival; AE, adverse events; MTD, maximum tolerated dose; IWG, International Working Group.

## FLT3 Inhibitors

FMS-like tyrosine kinase 3 (FLT3) is a transmembrane receptor expressed in CD34+ hematopoietic stem cells that plays a critical role in both proliferation and apoptosis through several key cellular signaling pathways including phosphatidylinositol-3-kinase and RAS (25). FLT3 internal tandem duplication mutation (FLT3-ITD) is found in approximately 25% of AML cases and is considered a high-risk feature (26, 27). Although allogeneic stem cell transplantation is utilized in the treatment of patients with FLT3-ITD AML, these patients have a higher incidence of relapse and decreased leukemia-free survival when compared to those with non-FLT3-ITD AML (28). With FLT3 mutation prevalence and prognostic impact in mind, several FLT3 inhibitors have been developed and utilized both pre- and post-transplant. These agents can be largely characterized in two categories: first generation/multi-targeted tyrosine kinase inhibitors (TKIs), and next generation/selective TKIs (27, 29).

Sorafenib is amongst the growing number of first generation TKIs with promising efficacy as post-transplant maintenance therapy in FLT3-ITD AML. To date, two large-scale randomized control trials have been published with data supportive of sorafenib use in this treatment setting (9, 10). As published by Xuan et al. in 2020, a phase III clinical trial recruited 202 patients with FLT3-ITD AML across seven hospitals in China and randomized patients at post-transplant day 30 to either placebo or sorafenib 400 mg twice per day until post-transplant day 180. They found that patients receiving sorafenib maintenance therapy had a cumulative 1-year incidence of relapse of 7.0% (95% CI 3.1%-13.1%) as compared to 25% (95% CI 16.6%-33.3%) in the placebo group (HR 0.25; 95% CI 0.11-0.57; p=0.0010) (9). There was no significant difference in the overall incidence of grade 3 or 4 adverse events between the sorafenib and placebo groups, though there was a relative increase in the incidence of grade 3 or 4 hematologic (15% sorafenib, 7% placebo) and dermatologic (7% sorafenib, 1% placebo) adverse events (9). *Post-hoc* multivariable analysis found that sorafenib maintenance therapy was the only protective factor in survival (9). Published within the same month, the SORMAIN trial recruited 83 patients with FLT3-ITD AML and randomized patients to 24 months of sorafenib maintenance therapy or placebo starting between post-transplant day 60 and 100 (10). Patients receiving sorafenib compared to placebo had a HR of relapse or death of 0.39 (95% CI 0.18-0.85; p=0.013), and 2-year relapse-free survival (RFS) of 85% (95% CI 0.70%-0.93%) compared to 53% in the placebo group (95% CI 0.36%-0.68%); the overall HR for relapse or death was 0.256 (95% CI 0.10-0.65; p=0.002) (10).

Midostaurin is another multi-targeted TKI with growing evidence in both the pre- and post-transplant treatment of FLT3-ITD AML (11, 30). The RADIUS trial was a phase II clinical trial randomizing 60 patients with FLT3-ITD AML after allogeneic stem cell transplant to up to 12 4-week cycles of standard of care treatment with or without midostaurin maintenance therapy (11). The 18-month RFS of patients with midostaurin was 89% (95% CI 69%-96%) as compared to 76% with standard of care alone (95% CI 54%-88%); rates of relapse were 11% and 24% respectively, resulting in a 46% relative reduction (11). It should be noted that the study was not powered to detect a statistically significant difference between the two arms of the trial, with the authors generating a sample size of 60 patients to detect a 50% reduction in the relative risk of relapse (11). Finally, multiple clinical trials are investigating the use of other broadly active TKIs in the post-transplant setting, such as ponatinib in the PONALLO trial (NCT03690115).

Numerous selective TKIs have been studied in the post-transplant maintenance of FLT3-ITD AML, though it remains too early for definitive conclusions regarding their efficacy in this treatment setting. Blood and Marrow Transplant Clinical Trials Network Protocol 1506 (NCT02997202) is a phase III randomized control trial with patients receiving gilteritinib or placebo after undergoing allogeneic stem cell transplant; the trial has completed patient accrual and is currently underway (12). Crenolanib is being evaluated in the single-arm phase II clinical trial NCT02400255 with two cohorts: patients in complete remission at time of transplant, and those that were not in complete remission at time of transplant. Quizartinib was the subject of a 2018 phase I clinical trial of 13 patients with FLT3-ITD AML which showed acceptable tolerability and only 1 patient experiencing relapse (13).

## Histone Deacetylase Inhibitors

Histone deacetylase inhibitors (HDACi) are agents which enact epigenetic change on oncogenes or tumor suppressor genes to elicit cell cycle arrest, cessation of cellular differentiation, and apoptosis (31). HDACi have been shown to have a variety of other potentially therapeutic effects, including increased reactive oxidative species and regulation of death receptor expression (32). Panobinostat, a non-selective HDACi, was shown in phase I clinical trial to have antileukemic effect in patients with high risk refractory AML, acute lymphocytic leukemia (ALL), and myelodysplastic syndrome (MDS) (33). The PANOBEST trial published by Bug et al. in 2017 was a phase I/II clinical trial demonstrating 2-year overall survival (OS) of 88% and RFS of 74% in patients receiving panobinostat after allogeneic stem cell transplant, which compared favorably to similar cohorts (14). An active phase III randomized control trial (NCT04326764) is comparing the combination of panobinostat and donor lymphocyte infusions to standard of care (donor lymphocyte infusions alone) after allogeneic stem cell transplant and monitoring survival over 5 years; 52 patients have been enrolled since July 2018, and the study has an estimated primary completion date of June 2022.

## IDH-1 and IDH-2 Inhibitors

Isocitrate dehydrogenase 1 and 2 (IDH1 and IDH2) are enzymes involved in the conversion of isocitrate to 2-oxoglutarate. The accumulation of this product can result in inhibition of histone demethylases and the downstream modified expression of oncogenes and tumor suppressor genes (34, 35). Occurring in approximately 20% of patients with AML, these mutations are generally associated with adverse effects on RFS, especially mutations to IDH2 (36, 37). Ivosidenib and enasidenib are first-in-class oral therapies which inhibit IDH-1 and IDH-2 respectively and have a growing role in the treatment of IDH-mutated AML (38–41). The active phase I clinical trial NCT03515512 has enrolled 23 patients with IDH2-mutated AML who received enasidenib after allogeneic stem cell transplant. As reported by Fathi et al. in 2020, enasidenib was well-tolerated without a report of dose-limiting toxicity and a relapse rate of 13% (with note that longer follow up is necessary for further insight) (15). Additional active clinical trials NCT03728335 and NCT04522895 are evaluating the use of enasidenib in the post-transplant setting. The phase I trial NCT03564821 is evaluating the use of ivosidenib in the post-transplant setting for patients with IDH1-mutated AML; enrollment began in January 2019 and the estimated primary completion date is December 2022.

## Hypomethylating Agents

Hypomethylating agents (HMAs) are another class of drugs that function through epigenetic manipulation. Azacitidine (AZA) and Decitabine (DAC) are nucleoside analogues that irreversibly bind to enzymes responsible for methylation and induce cellular degradation (42, 43). Given the overall suppressive effect of DNA methylation, the downregulation of tumor suppressor genes can have significant implications on apoptosis. As such, HMAs are used to enhance expression of these genes and induce cellular death. HMAs have become proven and effective components of the treatment of AML (26, 42, 44). Their use in the post-transplant treatment phase has been proposed due to induction of graft versus leukemia, increased NK cell activity, and accelerated reconstitution of Treg cells (45, 46). Additional studies have shown that they induce endogenous retroviral elements leading to interferon-mediated death of cancer cells (47). When first utilized in the 1960s and 1970s, HMAs were administered at high doses with unacceptable toxicities and insufficient anti-tumor effect; however, it was found that their anti-leukemic effect was not dose-dependent, and protocols incorporating lower doses at more frequent intervals both reduced toxicity and increased efficacy (48–50). Highlighting these promising effects, a large meta-analysis published by Bewersdorf et al. in 2021 studied 809 patients undergoing post-transplant maintenance therapy with either TKIs (for those with FLT-ITD AML) or HMAs with control groups receiving standard-of-care post-transplant therapy. 2-year OS rates were 81.7% (95% CI 73.8-87.7%) and 65.7% (95% CI 55.1-74.9%) among patients treated with TKIs and HMAs respectively (51).

Multiple studies have been conducted regarding the use of AZA in AML after allogeneic stem cell transplant, and several have shown efficacy as a salvage therapy (52, 53). As maintenance therapy, several observational and single arm trials have shown efficacy. Ali et al. performed a retrospective analysis from two separate institutions comparing AZA maintenance therapy post-transplant (n=59) with historical controls (n=90). Their data showed that AZA maintenance therapy improved event-free survival (EFS) (p=0.019) and OS (p=0.011) (54). Other studies show similar results, with tolerable toxicities and modest improvements in event free survival (EFS) and OS (16–18). Oran et al. recently published a phase III, open label-randomized trial with AZA maintenance therapy post-transplant in November 2020; unfortunately, this did not show any improvement in RFS for the 187 enrolled patients but did show a higher toxicity burden in the AZA maintenance arm (19).

Intravenous AZA maintenance therapy can be highly disruptive to post-treatment life due to the need for frequent infusion appointments as well as toxicities including cytopenias and diarrhea (18, 55). In recognition of these issues, an oral form of AZA (CC-486) has been developed. CC-486 has been able to limit toxicity while prolonging exposure to the drug and increasing its ability to amplify hypomethylation (20). The QUAZAR AML-001 phase III double blind, randomized control trial studied patients with AML who are in clinical remission, but not a candidate for transplant; it was published in December 2020 by Wei et al. and showed favorable results. The CC-486 treatment arm (n=238) had a significantly longer median OS of 24.7 months versus 14.8 months for placebo (p<0.001) (56). This was also demonstrated in RFS of 10.2 months with CC-486 versus 4.8 months with placebo (p<0.001) (56). Based on this trial, CC-468 has been FDA approved for maintenance for first remission after induction therapy in patients who were not candidates for transplant. This drug is being further investigated for its efficacy and tolerability in different populations with current clinical trials (NCT04887857, NCT04806906) both as monotherapy and in combination with other therapeutic agents. Excitingly, this drug has also been examined in the post-transplant maintenance setting. De Lima et al. recently published the first prospective phase I/II dose-finding study for CC-486 as post-transplant maintenance therapy in AML or MDS. Their trial studied 30 patients on 4 different CC-486 dosage schedules in repeated 28-day cycles: CC-486 200 mg daily for 7 days per cycle, 300 mg daily for 7 days per cycle, 150 mg daily for 14 days per cycle, or 200 mg daily for 14 days per cycle. The 1-year cumulative incidence of relapse was 43% in the combined 7-day dosing group versus 13% in the combined 14-day dosing group (20). Similar results were seen in the 1-year relapse and progression-free survival (PFS) rates, with 54% and 72% in the 7-day and 14-day dosing groups respectively (20). Treatment emergent adverse events were mostly gastrointestinal and hematologic events, with 22 patients (73%) experiencing grade 1-4 events, one of which (intracranial hemorrhage) resulted in death (20).

DAC is another HMA utilized for induction chemotherapy in AML that has growing evidence for its use as maintenance therapy (7, 21, 26, 57). A dose and frequency of DAC 10mg/m2/day for 5 days was proposed by Pusic et al. due to decreased hematologic toxicity compared to higher doses; however, relapse was seen in 6 of the 22 patients with this regimen (21). A later study by Ma et al. conducted between 2015 and 2018 had more favorable results with a regimen of DAC 20 mg/m2/day for 5 days every three months. The 3-year OS was 92.9% versus 51.8% (p=0.003) and the 3 year DFS was 94.1% versus 55% (p=0.002) comparing the DAC and control arms respectively (57).

## Azacitidine + Gemtuzumab Ozogamicin

Gemtuzumab ozogamicin (GO), a recombinant humanized monoclonal antibody conjugated to the cytotoxic antibiotic calicheamicin, is another exciting novel therapeutic agent being tested as post-transplant maintenance therapy in combination with AZA. The antibody component targets the CD33+ cell surface marker that is expressed on cancerous cells in the majority of AML patients (58). Once the antibody locates the CD33+ leukemic cell, the antibiotic is internalized, ultimately leading to cell death (59). Interestingly, GO was initially approved for the treatment of CD33+ AML in 2000 but voluntarily removed from the market after fatal adverse events including hemorrhage, infection, and acute respiratory distress syndrome were observed; it was approved once again by the FDA in 2017 with dose adjustment.

In 2014, Oshikawa et al. published a small study of 10 post-transplant patients started on maintenance therapy with intravenous AZA 30 mg/m2 days 1–7, followed by GO at 3 mg/m2 on day 8. This was repeated every 4 weeks, or as soon as the patient’s hematologic counts recovered. The study ultimately was unable to contribute any statistically significant data, though it reported an OS of 70% versus 59.8% (p=0.138) and DFS 60% versus 42.8% at 1 year (p=0.222) when comparing the groups receiving AZA and GO to control (58). Additional significant study limitations included the control group composition (were randomly chosen from the institution’s database as the “no maintenance” arm) (58). Data unfortunately remains limited on the combination of AZA and GO, perhaps in recognition of the aforementioned toxicity of GO.

## BCL2 Inhibitors

B Cell leukemia/lymphoma 2 (BCL2) is an oncoprotein that acts to promote cell survival and prevent apoptosis. Venetoclax is a BCL2 inhibitor which acts as a BCL2-homology 3 mimetic, binding the oncoprotein and allowing for appropriate cellular death (60, 61). It has been shown to be safe and very tolerable for patients with relapsed/refractory AML, or in patients who are not fit to receive intensive chemotherapy (61, 62).

Venetoclax has been used as monotherapy for maintenance post-transplant. In 2020, Kent et al. published the results of 23 post-transplant patients (22 AML and 1 MDS) who received venetoclax daily titrated to a final dose of 400 mg daily. 6-month OS and RFS were both 87% (63). The most commonly reported adverse effects were cytopenias and diarrhea, with 3 patients discontinuing the drug due to adverse events (63). Additional case series have demonstrated the reasonable tolerability and low toxicity of venetoclax both alone and in combination with additional agents (64, 65).

The combination of HMAs (namely AZA and DAC) and BLC2 inhibitors (venetoclax) is a treatment regimen of considerable recent interest and examination. Their use pre-transplant has grown in no small part due to their relative tolerance compared to traditional induction and consolidation regimens, prompting experimentation in the post-transplant setting (26, 66). A retrospective study from 11 German transplant centers evaluating 30 post-transplant MDS or AML patients with relapsed disease who received AZA or DAC with venetoclax showed an overall response rate (ORR) of 47%, with no significant difference seen when comparing those receiving AZA and venetoclax to those receiving DAC and venetoclax (67). Notably, 29 of the 30 patients had neutropenia and there was a 16% rate of fatal infections, highlighting the risks associated with these combinations (67).

The first prospective trial using DAC and venetoclax as post-transplant maintenance therapy was published in 2020 by Wei et al. 6 patients were studied, with 2 in partial remission prior to transplant and 4 with minimal residual disease (MRD). Approximately day 100 post-transplantation, all patients received DAC 15 mg/m2/day for 3 days followed by venetoclax 200 mg on days 1-21. Results were promising, with both 2-year OS and EFS of 83%; 33% of patients had grade 1-2 adverse events (most commonly neutropenia, thrombocytopenia, anemia, and neutropenic fever), and none experience grade 3 or 4 adverse events (22). Reporting again on this study in 2021, Wei et al. provided new data after recruiting 20 total patients (17 with AML, 3 with MDS). Incorporating these new patients, the 2-year OS and EFS were 85.2% and 84.7% respectively (23).

AZA and venetoclax is another combination being actively evaluated as post-transplant maintenance therapy. NCT04161885 is an active phase III randomized open-label trial evaluating AZA and venetoclax in post-transplant patients currently in clinical remission; enrollment began February 2020 with an estimated primary completion date of 2025. NCT04128501 is a phase II trial using AZA and venetoclax as maintenance therapy in patients with MRD after allogeneic stem cell transplant for AML, T-cell leukemia, and acute mixed type leukemia; this study is also actively recruiting and has an estimated primary completion date of October 2022.

## ICP Inhibitors

Immune checkpoints (ICP) are proteins that function to identify healthy cells to T regulatory cells in normal immune function (68). There are numerous IPCs of therapeutic significance, including cytotoxic T-lymphocyte- associated protein 4 (CTLA-4) and programmed death 1 (PD-1) receptors and their respective ligands (B7-1/B7-2 and PD-L1/PD-L2). Tumor cells manipulate this system by altering their expression of ICPs to appear as normal cells to the immune system, allowing them to escape destruction (68). ICP inhibitors function to block this escape mechanism, allowing for proper immunologic function and cellular destruction; in this way, ICP inhibitors work to increase the graft versus leukemia effect. Examples of ICP inhibitors include the monoclonal antibodies nivolumab (anti-PD1), ipilimumab (anti-CTLA-4), and pidilizumab (anti-Delta-like 1).

Limited studies have been published regarding the use of ICP inhibitors in AML; however, there are numerous clinical trials underway evaluating their safety and efficacy in this application. Nivolumab has been shown to be a successful maintenance therapy in patients with high-risk AML in remission who are not candidates for allogeneic stem cell transplant (24). A 2021 phase II clinical trial of 6 measurable residual disease (MRD) negative patients and 9 MRD positive patients receiving nivolumab showed only 1 MRD negative patient experiencing relapse but only 2 patients with MRD positive AML achieving remission; this study was not in support of single use agent nivolumab but encouraged future directions (24). A 2016 phase I/Ib clinical trial by Davids et al. studied ipilimumab in AML patients with relapsed disease post-transplant, with 5 patients (22%) achieving a complete response (69). However, significant immune-related side effects were observed in 6 patients, with 4 developing graft versus host disease (GvHD) (69). The REMAIN trail (NCT02275533) seeks to assess single agent nivolumab as maintenance therapy post-induction and consolidation chemotherapy. NCT02846376 is an active trial investigating the role of nivolumab and ipilimumab as post-transplant maintenance therapy in 8 AML patients at high risk of relapse; it has an estimated completion date of December 2023.

In addition to ICP inhibitors alone as post-transplant maintenance therapy, there are trials underway to further assess the synergistic relationship between HMAs and ICP inhibitors. Treatment with HMAs have been shown to increase tumor cell expression of ICPs such as PD-1, PD-L1, PD-L2, and CTLA-4 (70). This has been seen in MDS, AML, and chronic myelogenous leukemia (CML), as demonstrated by Yang et al. in 2014 with the use of DAC (70, 71). Thus, the combination of HMAs and ICP inhibitors seeks to theoretically use HMAs to increase ICP expression which can be subsequently targeted with ICP inhibitors. This is a relatively new direction in AML treatment, and there is still much more to be studied regarding the combination of these drugs. One active but no longer recruiting clinical trial (NCT02775903) is utilizing AZA and durvalumab (anti-PDL1) in high-risk MDS and elderly patients with AML.

## Conclusion

Despite the advent of allogeneic stem cell transplant and the monumental change it brought to the treatment of AML, mortality remains high even amongst patients well enough to undergo transplant. Relapse after stem cell transplant can be devastating and highlights the need for treatment modalities to increase disease-free survival. New and innovative advancements in maintenance therapies has the potential to improve both overall survival and disease-free survival in this patient population, with the added potential for toxicity. Continued evidence is arising for maintenance treatment with varying combinations of the previously discussed therapies, as their largely favorable side effect profiles make them desirable. Post-transplant maintenance therapy is a new frontier of AML treatment and will continue to expand with further research.

## Author Contributions

SM and TR were responsible for the citation research, data analysis, and prose writing for the entirety of this review. NP and LW provided the topic of discussion, initial foundational research and guidance, extensive content review and editing, and final approval for submission.

## Conflict of Interest

The authors declare that the research was conducted in the absence of any commercial or financial relationships that could be construed as a potential conflict of interest.

## Publisher’s Note

All claims expressed in this article are solely those of the authors and do not necessarily represent those of their affiliated organizations, or those of the publisher, the editors and the reviewers. Any product that may be evaluated in this article, or claim that may be made by its manufacturer, is not guaranteed or endorsed by the publisher.
